# Nutritional and Feeding Adaptability of *Clanis bilineata tsingtauica* Larvae to Different Cultivars of Soybean, (*Glycine max*)

**DOI:** 10.3390/foods12081721

**Published:** 2023-04-20

**Authors:** Lei Qian, Bo-Jian Chen, Fu-Rong Gui, Yi Qin, Pan Deng, Huai-Jian Liao

**Affiliations:** 1 Institute of Leisure Agriculture, Jiangsu Academy of Agricultural Sciences, Nanjing 210014, China; 2 College of Haide, Ocean University of China, Qingdao 266100, China; 3 State Key Laboratory of Conservation and Utilization of Biological Resources of Yunnan, College of Plant Protection, Yunnan Agricultural University, Kunming 650500, China; 4 College of Biotechnology, Jiangsu University of Science and Technology, Zhenjiang 212100, China

**Keywords:** *Clanis bilineata tsingtauica*, edible insects, nutrients, *Glycine max*, plant VOCs, host selection

## Abstract

The larvae of *Clanis bilineata tsingtauica*, a special species of Chinese edible insect, are of great nutritional, medicinal and economic value to humans. This study aimed to clarify the effect of different soybean varieties (Guandou-3 (G3), Ruidou-1 (R1), September cold (SC)) on the nutritional quality and feeding selection behavior of *C. bilineata tsingtauica* larvae. The results showed that soybean isoleucine (Ile) and phenylalanine (Phe) were positively correlated with larval host selection (HS) and protein content. The order of soybean plants selected by *C. bilineata tsingtauica* larvae was R1 > SC > G3, and they selected R1 significantly higher than SC and G3 by 50.55% and 109.01%, respectively. The protein content of the larvae fed on R1 was also the highest among the three cultivars. In addition, a total of 17 volatiles belonging to 5 classes were detected from soybeans: aldehydes, esters, alcohols, ketones, and heterocyclic compounds. Pearson’s analysis showed that soybean methyl salicylate was positively correlated with larval HS and their protein content, and soybean 3-octenol was negatively correlated with larval HS and their palmitic acid content. In conclusion, *C. bilineata tsingtauica* larvae are more adapted to R1 than to the other two soybean species. This study provides a theoretical basis for the production of more protein-rich *C. bilineata tsingtauica* in the food industry.

## 1. Introduction

In recent years, the issue of global food security, first mentioned by Meyer-Rochow [1], mainly due to climate change and human activities, has received increasing attention from governments and scientists [2,3]. Edible insects are a potential sustainable source of animal protein and can meet the growing global demand for new protein sources [4,5]. They can provide the same amount of protein as cattle, pigs, and poultry, while using less land and water and producing much lower levels of greenhouse gases [6,7]. *Clanis bilineata tsingtauica* belongs to the family Sphingidae of Lepidoptera and is a unique species of edible Chinese insect, and its production has become a characteristic agricultural industry in rural Chinese areas [8]. Its larvae are rich in proteins, essential amino acids (EAAs), unsaturated fatty acids (UFAs; highly valuable compounds in the food industry), vitamins, trace elements, chitin, and lecithin [9]. These nutritional components (mainly proteins, EAAs, UFAs, and trace elements) can promote brain development, prevent cell deterioration and maintain human endocrine balance [9,10]. The artificial breeding of *C. bilineata tsingtauica* has become a special agricultural industry in China, with an annual production of 30,000 tons and a value of nearly 4.5 billion RMB [11]. However, the nutrition of *C. bilineata tsingtauica* is affected by pests, diseases, photoperiod, and weather, and there is a great challenge to provide safe, stable, and nutritious larvae for the food industry. First, we need to understand the feeding adaptations of *C. bilineata tsingtauica* to its host plants, and then further clarify the factors that affect its feeding and nutritional quality.

Phytophagous insects decide whether or not to feed on host plants based on the amount and type of nutrients present in the plants [12]. The abundance and appropriate levels of nutrients are crucial for the growth, development, and nutritional quality of insects [13,14]. Sugar is not only an important source of energy metabolism in insects but also acts as a feeding stimulant that affects the life activities of herbivorous insects. Adequate levels of plant sugars can increase the food intake of cotton bollworms (*Helicoverpa armigera*) and promote their growth and development, pupal weight, and also nutritional quality [15]. However, sucrose content in poplar (*Populus* L.) leaves was found to correlate negatively with the growth and development of fall webworms (*Hyphantria cunea*) [16]. Amino acids are a class of bioactive substances and crucial nutrients for insects that perform multiple functions, such as participating in energy metabolism, protein and fatty acid synthesis, and regulation of blood osmolality. Some insects cannot synthesize certain amino acids (EAAs), which are usually obtained from diets (mainly host plants) to support their growth and development [17,18]. Low levels of amino acids in plants or unbalanced relative ratios of different amino acids can affect insect growth and development. Feeding on rice plants with higher nitrogen content increases the survival rate and shortens the developmental period of the brown planthopper (*Nilaparvata lugens*) [19]. Protein is an essential food source for phytophagous insects and the best nutrient for their growth and development. Insects prefer to feed on host plants with high protein content so that the larvae can still get enough nutrients with limited food to achieve their growth and development, improve food utilization, conversion rate, etc. [20]. Although some studies on the nutritional status of *C. bilineata tsingtauica* larvae have been reported [9,21], the adaptive mechanism of larval nutrition and feeding behavior to different hosts is largely unexplored.

Most insects depend on the major nutrients (e.g., proteins and carbohydrates) in plants for their growth and development, and plants in turn have evolved to produce abundant secondary metabolites in response to phytophagous insect feeding. Insects achieve the balance to ensure their growth and development by feeding on and absorbing nutrients from host plants and adapting to the metabolism of plant secondary metabolites [22,23]. Sensitive olfactory systems allow insects to detect chemical signals in the environment [24,25]. For example, the emission of volatile organic compounds (VOCs) involved in plant defense can alter insect host selection behavior and attract carnivores (usually arthropod predators or parasitoids) to prey on or parasitize the insects, thereby reducing herbivore pressure on damaged plants [26,27]. Some phenolic compounds found in many plants can prevent feeding, reduce digestion, and produce toxins for insects [28]. Elevated levels of flavonoids have been found in insects that feed on poplar, which may disrupt the insects’ normal metabolic process of repelling or resisting ingestion [29,30].

To understand the nutritional and feeding adaptability of *C. bilineata tsingtauica* larvae to different soybean cultivars, this study investigated the variation of nutrients and plant VOCs among three soybean cultivars and how they affect the larval nutrient quality and host selection behavior. We hypothesized that (i) plant nutrients directly alter the host selection behavior and nutrient levels of *C. bilineata tsingtauica* larvae and (ii) some major VOCs from soybeans will repel or attract insects by altering their feeding selection behavior, thereby indirectly affecting the nutritional quality of insects. The research results provide a scientific basis for producing higher-protein *C. bilineata tsingtauica* larvae and a theoretical foundation for the industrialization and commercialization of the *C. bilineata tsingtauica* industry.

## 2. Materials and Methods

### 2.1. Soybean Cultivars

Three common field-used cultivars of soybean—*Glycine max* L. (Guandou-3 (G3), Ruidou-1 (R1), and September cold (SC))—were obtained from Yuntai Farm of the Jiangsu Agricultural Reclamation Group Co., LTD (Lian Yun-gang, Jiangsu, China; 119.29° E, 34.59° N). The seeds were sown in flowerpots (32 cm × 23 cm) with 1.5 kg of soil (Xinggong Organic Fertilizer Co., Ltd., Zhenjiang, China) in an artificial climate chamber (ACC) (RXZ-380, Ningbo Southeast Instruments Co., Ltd., Ningbo, China) in the Laboratory of Insect Resource Utilization at the Jiangsu Academy of Agricultural Sciences (Nanjing, Jiangsu, China; 118.88° E, 32.04° N). The flowerpots were randomly rotated once every two days to minimize the position effect. No fertilizer or pesticide was used during the growing period of soybean plants. The ACC was set to temperature 26 ± 1 °C, relative humidity (RH) 70 ± 5%, photoperiod light:dark 16:8 h, and light intensity 20,000 Lx.

### 2.2. Insect Stocks

A population of *C. bilineata tsingtauica* was initially established from eggs provided by Yuntai Farm. The newly emerged adults were mated and kept in nylon cages with hydromel for egg laying. Nylon nets containing the egg masses were kept in rectangular plastic boxes with moist absorbent cotton until the eggs hatched. Freshly emerged larvae were kept in the insectary with soybean leaves under conditions of 16 h light, 26 ± 1 °C, and 70 ± 5% RH and 8 h dark, 25 ± 1 °C, and 70 ± 5% RH. Soybean leaves were replaced daily.

### 2.3. Measurement of Nutrients in Soybeans

Fresh leaves were randomly collected on the 35th day after the sowing of soybean seeds (G3, R1, SC) for the measurement of nutrient content. Half a gram of fresh leaflets and phosphate-buffered saline (PBS, pH 8.0) was placed in a mortar, ground with a pestle in an ice-water bath, and centrifuged at 2500× *g* for 10 min. The supernatant was used as the test solution. The composition and content of amino acids in soybeans were determined by a Hitachi amino acid analyzer using EZChrom Elite software (L-8900, Hitachi Limited, Japan). The content of soluble protein and soluble sugar in soybeans was determined using a total protein quantification kit and plant soluble sugar content test kit) from Qingdao Sci-Tech Innovation Quality Testing Co. LTD (Qingdao, China). The optical density (OD) of the reactants was measured using an ultraviolet spectrophotometer (UV-1800PC, Mapada; Shanghai, China). Three replicates per soybean variety were set up.

### 2.4. Analysis of Plant VOCs from Soybeans

The dynamic headspace adsorption method was used to collect plant volatile organic compounds (VOCs) from three soybean varieties. The apparatus includes an air pump, flow meter, wash bottle, drying tower, bottle, and adsorption tubes. The reagent consumables used include n-hexane and Tenax (200 nm), screw-mouth sample bottle (4 mL), brown screw-mouth automatic sample injection bottle (9 mm), and high-purity nitrogen. The composition and content of plant VOCs from soybeans were measured by gas chromatography–mass spectrometry (GC-MS, 320-MS; Brook Dalton mass spectrometry Co., Dalton, MA, USA). The GC was equipped with an Agilent HP-5 capillary column, and the injector temperature was set to 250 °C. Helium was used as the carrier gas at an average flow rate of 1.0 mL/min. The MS method was as follows: ionization mode EI 70 eV, source and transfer line both kept at 230 °C, and scanned area 50–550 *m*/*z*. Three replicates per soybean variety were set up.

### 2.5. Host Selection Assays of C. bilineata tsingtauica on Soybean

A four-chamber olfactometer (PSM4-150; Nanjing, China) was used to analyze host selection of *C. bilineata tsingtauica* fifth-instar larvae on soybean. Three soybean cultivars were randomly selected and placed in each arm of the olfactometer, while the fourth arm was sealed with plastic wrap. An 8 W fluorescent lamp was placed over the four-arm motherboard and the flow meter was adjusted to provide a uniform airflow of 3 L/min on all three sides. Fifteen fifth-instar larvae were randomly selected to be starved for 6 h and then released into the center of the four-armed motherboard to observe their host selection behavior. If the larvae reached the nesting area of an arm within 20 min, the treatment corresponding to that arm was considered the choice of the released *C. bilineata tsingtauica*. Those larvae that did not reach either side of the nest within 20 min of release were considered nonresponders (i.e., no choice). To avoid bias in behavioral observations between trials, the air compressor was turned off for 10 min after each trial and wiped down with anhydrous alcohol. The intake tube was also replaced after each trial, and all trials were conducted in a clean, uniform, well-ventilated, and relatively enclosed laboratory. The details of measuring host selection behavior were based on Qian’s methods [31]. Three replicates per soybean variety were set up.

### 2.6. Measurement of Nutrients in C. bilineata tsingtauica

Fresh soybean leaves (G3, R1, SC) were randomly selected and wrapped around the petiole with moistened absorbent cotton to prevent wilting. The leaves were placed in a petri dish, 10 newly hatched *C. bilineata tsingtauica* larvae were added, and the dish was sealed with plastic wrap and cling film. The plastic was changed once a day. When the larvae grew to the fifth instar, they were collected for a 12 h starvation treatment to eliminate food in the larval gut. The fresh larvae were then prepared for nutrient measurements.

The crude protein content of *C. bilineata tsingtauica* larvae was determined using an automatic Kjeldahl apparatus (K9840; Jinan Haineng Instrument Co., Jinan, China). The fresh sample (0.2 g) was mixed with copper sulfate, potassium sulfate, and sulfuric acid for slaking. When the temperature of the slaking furnace reached 420 °C, slaking was continued for 1 h. It was taken out and cooled when the liquid became clear and transparent, and 20 mL of distilled water was added for 7 min on the automatic Kjeldahl apparatus. It was mixed with two drops of indicator mixture solution and 10 mL of boric acid, the distillate solution was made up to 200 mL, then titrated with hydrochloric acid (HCl) standard solution (0.10 mol/L). The endpoint was light gray-red and a reagent blank was made simultaneously.

The composition and content of larval Asp, Thr, Ser, Glu, Gly, Ala, Cys, Val, Met, Ile, Leu, Tyr, Phe, Lys, His, Arg, and Pro were determined using the Hitachi amino acid analyzer. The HCl solution was added to the ground samples, frozen for 5 min, and filled with nitrogen. Hydrolyzed at 110 °C in an electric blast thermostat for 22 h, and quantified to 50 mL. Filtrate of 1.0 mL was drawn off, decompressed at 40 °C and dissolved with sodium citrate buffer solution (1.0 mL, PH 2.2). The filtrate was filtered through a 0.22 μm membrane and then tested on the instrument. Trp levels were measured by high-performance liquid chromatography (HPLC) (1260, Agilent, Santa Clara, CA, USA). The standard curve was generated to ensure accuracy before testing the sample (Figure S1). Using an Agilent C18 capillary column for HPLC, the column temperature was set to 35 °C, and the injection volume was 10 μL at a flow rate of 1.2 mL/min.

The composition and content of fatty acids in *C. bilineata tsingtauica* larvae were analyzed by GC-MS. An Agilent HP-88 capillary column was used and the injector temperature was set to 290 °C. Helium was used as the carrier gas at a rate of 1.0 mL/min. The ionization mode was set to EI 70 eV, the source and transfer line were both kept at 280 °C, and the scanned area reached 30–400 *m*/*z*. Three replicates per soybean variety were set up.

### 2.7. Statistical Analysis

Data were analyzed using SPSS 25.0 software (Chicago, IL, USA). All measured index values are expressed as mean ± SEM (standard error of the mean). Nutrients and VOCs of soybean, the host selection of *C. bilineata tsingtauica* larvae for different soybean cultivars, and the content of protein, amino acids, and fatty acids of *C. bilineata tsingtauica* were analyzed by one-way analysis of variance (ANOVA). Significant differences between treatments were determined by Tukey test at *p* < 0.05. Correlations between soybean nutrients and VOCs and larval host selection and nutrients were gained by Pearson correlation analysis. Heat maps were generated using RStudio software.

## 3. Results

### 3.1. Effect of Nutrients in Different Soybean Cultivars on Host Selection and Nutrient Levels of C. bilineata tsingtauica

#### 3.1.1. Nutrient Levels in Three Soybean Cultivars

**Figure 1:**
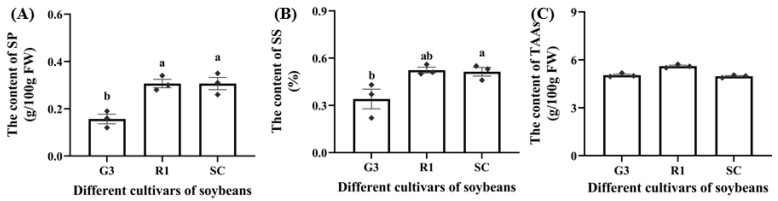
The content of soluble protein (**A**), soluble sugar (**B**) and total amino acids (**C**) in different soybean cultivars (G3, R1, SC). Data are means ± SEM. The lowercase letters in the figure indicate significant differences among different cultivars (*p* < 0.05, Tukey test). FW: fresh weight; SP: soluble protein; SS: soluble sugar; TAAs: total amino acids.

Three soybean cultivars (G3, R1, SC) differed in nutrient levels (Figure 1). Soluble protein (SP) of soybeans was extracted in PBS and measured using the quantification kit. The SP content in G3 was significantly lower than that in R1 and SC by 49.04% and 48.62%, respectively (*p* < 0.05, Figure 1A). The soluble sugar (SS) content in G3 was significantly lower than that in SC by 32.67% (*p* < 0.05, Figure 1B). In addition, the content of total amino acids (TAAs) was not significantly different among G3, R1, and SC (Figure 1C).

Although there were no significant changes in TAAs among the three cultivars, the individual amino acids were not consistent (Table 1). The contents of all amino acids in soybean are listed in Table 1. Glutamic acid (Glu) content was 28.74% higher in G3 than in SC (*p* < 0.05, Table 1). Cysteine (Cys) content was 23.60% higher in R1 than in SC (*p* < 0.05, Table 1). In addition, isoleucine (Ile) and phenylalanine (Phe), both EAAs for humans, showed a consistent variation trend in three soybean cultivars. The content of Ile and Phe in G3 was significantly lower than that in R1 by 12.34% and 24.36%, respectively (*p* < 0.05, Table 1).

#### 3.1.2. Effect of Nutrients in Different Soybean Cultivars on Host Selection of *C. bilineata tsingtauica*

**Figure 2:**
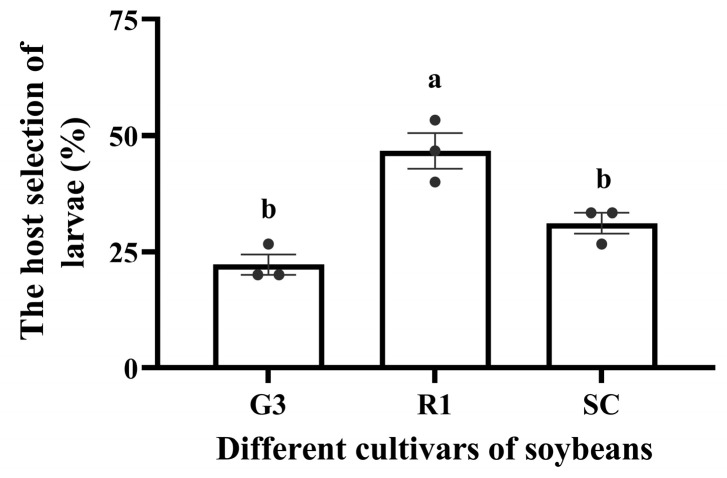
Host selection of *C. bilineata tsingtauica* larvae for different soybean cultivars. Data are means ± SEM. The lowercase letters in the figure indicate significant differences among different cultivars (*p* < 0.05, Tukey test).

**Figure 3:**
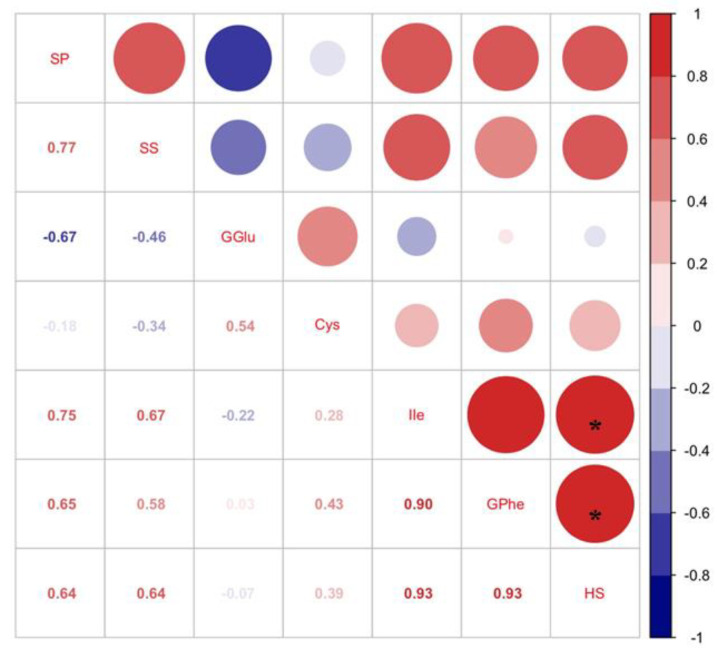
Pearson correlations between nutrients in soybean and host selection of *C. bilineata tsingtauica* larvae (SP: soluble protein; SS: soluble sugar; GGlu: glutamic acid in soybeans *G. max*; Cys: cysteine; Ile: isoleucine; GPhe: phenylalanine in *G. max*; HS: host selection of *C. bilineata tsingtauica* for soybean). The size of the blocks corresponds to the values in the figure, which represent the correlations between key plant VOCs and larval host selection and nutrients. Red blocks indicate positive correlations, blue blocks indicate negative correlations, and the asterisks within the blocks indicate significant correlations at *p* < 0.05.

According to the four-arm olfactometer test, the order in which *C. bilineata tsingtauica larvae* chose was R1 > SC < G3. The larvae selected R1 more than G3 and SC by 109.01% and 50.55%, respectively (*p* < 0.05, Figure 2). Pearson’s analysis showed that IIe and Phe in soybean plants were positively correlated with host selection of *C. bilineata tsingtauica* (*p* < 0.05, Figure 3).

#### 3.1.3. Effect of Nutrients in Different Soybean Cultivars on the Nutrient Content of *C. bilineata tsingtauica*

**Figure 4:**
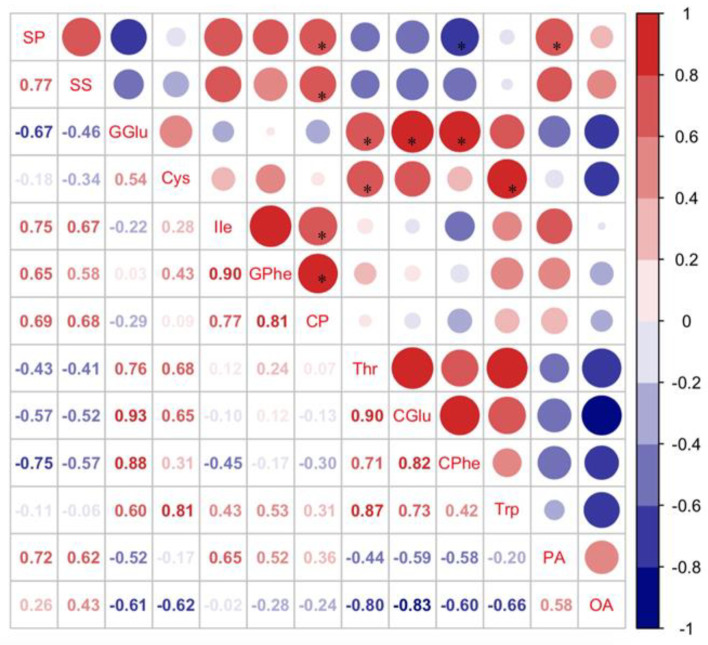
Pearson correlation between nutrients in soybean and nutrient content of *C. bilineata tsingtauica* larvae (CP: crude protein; Thr: threonine; CGlu: glutamic acid in *C. bilineata tsingtauica*; CPhe: phenylalanine in *C. bilineata tsingtauica*; Trp: tryptophane; PA: palmitic acid; OA: oleinic acid). The size of the blocks corresponds to the values in the figure, which represent the correlations between key plant VOCs and larval host selection and nutrients. Red blocks indicate positive correlations, blue blocks indicate negative correlations, and the asterisks within the blocks indicate significant correlations at *p* < 0.05.

**Figure 5:**
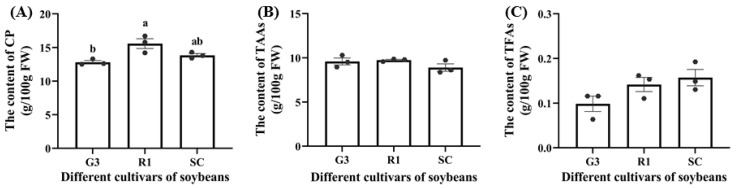
The content of crude protein (CP) (**A**), total amino acids (TAAs) (**B**) and total fatty acids (TFAs) (**C**) in *C. bilineata tsingtauica* larvae fed on different soybean cultivars. Data are means ± SEM. The lowercase letters in the figure indicate significant difference among different cultivars (*p* < 0.05, Tukey test). FW: fresh weight.

Crude protein (CP), total amino acids (TAAs), and total fatty acids (TFAs) in *C. bilineata tsingtauica* larvae were measured to clarify the effect of different soybeans on larval nutrition. Pearson’s analysis showed that there was a positive effect of soybean nutrients on *C. bilineata tsingtauica* larvae (Figure 4). Soluble protein (SP), soluble sugar (SS), Ile and Phe in soybean were all positively correlated with larval CP (*p* < 0.05, Figure 4). The CP content of larvae fed on R1 was significantly higher than that of larvae fed on G3 (*p* < 0.05, Figure 5A). This could be due to the higher nutrition in R1. Combining the selection result that *C. bilineata tsingtauica* larvae preferred R1 the most with the larval nutrition preference, the larvae were considered better adapted to R1.

Additionally, although there was no significant variation in larval TAAs (Figure 5B), the individual amino acids were different (Table 2). The contents of all amino acids in *C. bilineata tsingtauica* are listed in Table 2. Compared with those fed on SC, the levels of threonine (Thr), glutamic acid (Glu), phenylalanine (Phe), and tryptophan (Trp) in larvae fed on G3 were significantly higher by 20.18%, 21.85%, 29.48% and 24.33%, respectively (*p* < 0.05). Moreover, Pearson’s analysis showed that plant Glu and Cys were positively correlated with larval Thr (*p* < 0.05, Figure 4). Soybean Glu was also positively correlated with larval Glu and Phe (*p* < 0.05).

Total fatty acids (TFAs) were measured in *C. bilineata tsingtauica* larvae (Figure 5C), but only palmitic acid (PA) and oleic acid (OA) differed among the three soybean cultivars (Table 3). The PA content in *C. bilineata tsingtauica* larvae fed on G3 was significantly lower than those fed on R1 and SC by 48.26% and 49.53%, respectively (*p* < 0.05, Table 3). Pearson’s analysis showed that soybean SP was positively correlated with larval PA (*p* < 0.05, Figure 4). OA content in larvae fed on SC was significantly higher than those fed on G3 and R1 by 132.45% and 124.86%, respectively (*p* < 0.05, Table 3). The contents of all fatty acids in *C. bilineata tsingtauica* are listed in Table 3.

### 3.2. Effect of Plant VOCs in Different Soybean Cultivars on Host Selection and Nutrients in C. bilineata tsingtauica

#### 3.2.1. Plant VOCs in the Three Soybean Cultivars

A total of 17 volatiles belonging to five classes were detected in soybean plants, including aldehydes, esters, alcohols, ketones, and heterocyclic compounds. Aldehydes were the most abundant (six species in total). And benzaldehyde, nonanal, methyl salicylate, 3-octenol, 5, 6-epoxy-β-ionone, furan, 2-ethyl-, and furan, 2-pentyl- were all detected in G3, R1 and SC. In addition, heptadienal was detected in both G3 and SC, and salicylaldehyde, 1-penten-3-ol and trans-2-pentene were detected in both R1 and SC (Table 4).

**Figure 6:**
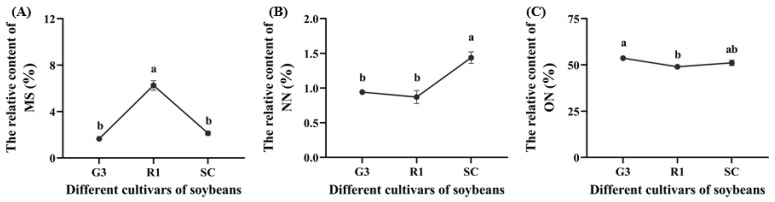
The relative content of methyl salicylate (MS) (**A**), nonanal (NN) (**B**) and 3-octenol (ON) (**C**) in different soybean cultivars. Data are means ± SEM. The lowercase letters in the figure indicate significant differences among different cultivars (*p* < 0.05, Tukey test).

The relative content of methyl salicylate (MS), nonanal (NN) and 3-octenol (ON) in soybeans varied significantly among G3, R1 and SC (*p* < 0.05, Figure 6). MS content in R1 was significantly higher than that in G3 and SC by 278.79% and 193.88%, respectively (*p* < 0.05, Figure 6A), NN content in SC was significantly higher than that in G3 and R1 by 52.84% and 65.14%, respectively (*p* < 0.05, Figure 6B), and ON content in G3 was higher than that in R1 by 9.43% (*p* < 0.05, Figure 6C). MS, NN and ON belong to the class of esters, aldehydes and alcohols, respectively.

#### 3.2.2. Effect of VOCs in Different Cultivars of Soybeans on Host Selection and Nutrients of *C. bilineata tsingtauica* Larvae

**Figure 7:**
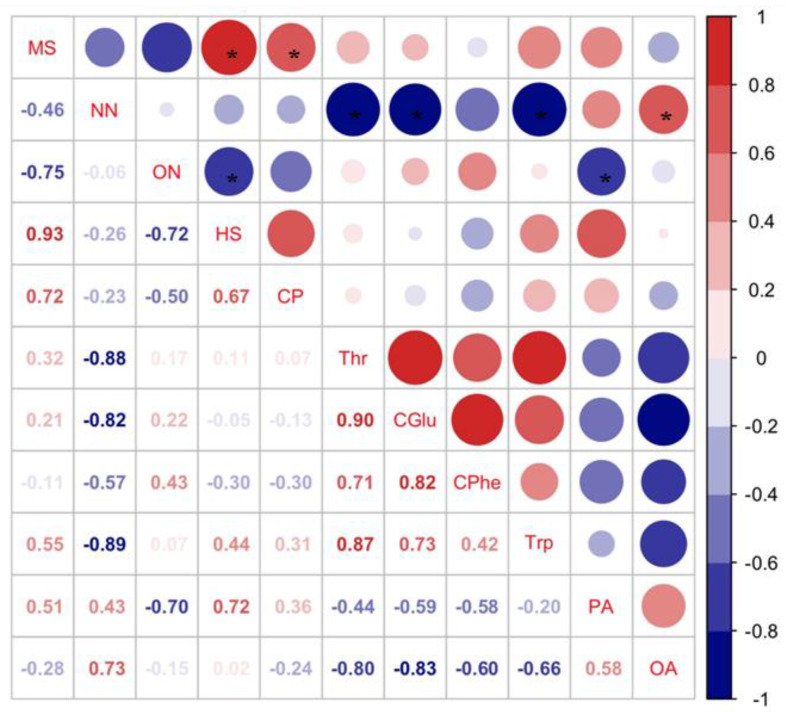
Pearson correlations between key VOCs in soybean and host selection and nutrients of *C. bilineata tsingtauica* larvae (MS: methyl salicylate; NN: nonanal; ON: 3-octenol; HS: host selection; CP: crude protein; Thr: threonine; CGlu: glutamic acid in *C. bilineata tsingtauica*; CPhe: phenylalanine in *C. bilineata tsingtauica*; Trp: tryptophane; PA: palmitic acid; OA: oleinic acid). The size of the blocks corresponds to the values in the figure, which represent the correlations between key plant VOCs and larval host selection and nutrients. Red blocks indicate positive correlations, blue blocks indicate negative correlations, and the asterisks within the blocks indicate significant correlations at *p* < 0.05.

Pearson’s analysis showed that host selection (HS) of *C. bilineata tsingtauica* larvae was positively correlated with methyl salicylate (MS) from soybean, but negatively correlated with soybean 3-octenol (ON) (*p* < 0.05, Figure 7). This indicated that *C. bilineata tsingtauica* larvae were sensitive to changes in MS and ON, which would affect larval feeding adaptation to host plants.

The relative content of MS from R1 was the highest among the three cultivars (Figure 7), but the content of ON from R1 was the lowest (Figure 6), supporting our finding that *C. bilineata tsingtauica* larvae were more adapted to R1 than the other two species. Soybean MS was positively correlated with crude protein (CP) in *C. bilineata tsingtauica* larvae. Soybean ON was negatively correlated with larval palmitic acid (PA) (*p* < 0.05, Figure 7). In addition, soybean nonanal (NN) was negatively correlated with larval Thr, Glu and Trp, but positively correlated with their oleinic acid (OA) (*p* < 0.05, Figure 7).

## 4. Discussion

Plant nutrients play an important role in the growth, development and nutritional quality of insects. This study indicates that soybean nutrients might improve the nutrition of *C. bilineata tsingtauica* larvae. Guo et al. [11] also found that plant nutrients were positively correlated with insect nutrition. Similarly, the western flower thrip (*Frankliniella occidentalis*) prefers plant pollen to leaves because of the higher protein content [32]. *Bradysia cellarum* and *Bradysia impatiens* also showed a nutrient preference for the chives and B-beans with higher levels of protein, amino acids, SS and starch than other plants. The contents of SS, lipid and protein were significantly higher in the two Bradysia species fed on chives and B-beans than in those fed on cabbage, lettuce, W-cabbage and pepper [33]. In addition, Glu in poplar leaves inhibits the growth and development of *H. cunea* [16]. Dietary supplementation of Glu shortened pupal duration and increased pupal weight in honey bees (*Apis mellifera*) [34]. The amino acids in soybean plants and *C. bilineata tsingtauica* larvae were both measured using an amino acid analyzer, including cysteine, which was fully recoverable by the analyzer. We speculated that the content of Thr, Glu, and Phe in the insects could be increased by regulating the Glu in the host plants, thus contributing to the improvement of overall amino acid levels in *C. bilineata tsingtauica*. Moreover, studies have shown that PA and OA are the predominant fatty acids in some edible insects (e.g., *Hermetia illucens*, *Polistes* sp., *Dactylopius* sp., *Euschistus taxcoensis*, *Euschistus strenuus*, etc.) [35,36,37]. OA has a beneficial effect on blood vessels reducing the risk of cardiovascular disease and is a precursor of α-linolenic acid and γ-linolenic acid, both of which are essential fatty acids (EFAs) [38]. Therefore, it is of great importance to produce a greater quantity of higher-EFA *C. bilineata tsingtauica* larvae for the food industry.

Insects mostly select host plants that meet their nutritional criteria to meet their physiological needs. Some insects with piercing and sucking mouthparts, such as aphids and leafhoppers, have a test-feeding habit. If the plants cannot provide the food they need, they will move to other plants to feed [39,40]. Some insects with chewing mouthparts probe the plants through receptors before deciding whether to stay on the plant. The gypsy moth (*Lymantria dispar*) has been reported to prefer plants with high nitrogen content [41,42]. However, *Liriomyza sativae* preferred plants with moderate nitrogen content, and its selection for bean plants decreased significantly with increasing potassium concentration [43]. It has also been reported that plants with lower amino acid content are generally more resistant to insects and diseases [27,44,45]. This is consistent with our finding that *C. bilineata tsingtauica* larvae selected the least for G3, which contains the lowest IIe and Phe. Furthermore, we speculated that some EAAs (e.g., IIe and Phe) could not be synthesized in insects or, to a lesser extent, must be obtained from host plants. Experiments with the exogenous addition of these EAAs are still needed to verify this point in the future. These results support our first hypothesis and provide a theoretical basis for the production of more protein-rich *C. bilineata tsingtauica*.

In addition to the nutritional effects of plants on insects, the emission of volatile organic compounds (VOCs) involved in ecosystem information transfer plays a vital role in insect behavior and physiological regulation, including attraction, antifeedant, growth inhibition and reproduction [23,27,46]. Many studies have shown that plant VOCs are important chemical signals for herbivorous insects to communicate with their host plants [47,48]. For example, parasitoid females are attracted to VOCs emitted by yarrow [49]. Huang et al. [50] also found that changes in plant VOCs directly transferred Lyonetia prunifoliella to different host plants and stimulated its adaptability (recognition and experience) to other plants. In this study, *C. bilineata tsingtauica* larvae were more adapted to R1, which has a higher content of MS and lower content of ON than the other two species. We speculated that soybean MS may attract *C. bilineata tsingtauica* larvae feeding on the plant, but ON may repel larval ingestion. Studies have reported that plant nutritional components can interact with secondary metabolites to affect insect performance and fitness [51,52]. This suggests that soybean emits VOCs that change the feeding adaptation of *C. bilineata tsingtauica* larvae, thereby altering their nutritional quality. This provides direct evidence for our second hypothesis. Studying on the function of major VOCs from host plants and finding out how to improve the nutritional value of edible insects by regulating the VOCs will contribute much to the *C. bilineata tsingtauica* industry.

## 5. Conclusions

The effect of plant nutrients and VOCs on the nutritional and feeding adaptability of *C. bilineata tsingtauica* larvae was investigated in this study. Overall, soybean nutrients have positive effects on the host selection (HS) and nutritional quality of *C. bilineata tsingtauica* larvae. The larvae selected R1 more than G3 and SC, and the protein content of the larvae fed on R1 was also the highest among the three soybean cultivars. In addition, soybean VOCs directly affect the HS of larvae, thereby altering the nutrition of *C. bilineata tsingtauica*. A total of 17 volatiles belonging to 5 classes were detected from soybeans. Soybean methyl salicylate may attract *C. bilineata tsingtauica* larvae to feed on and increase their protein content. However, soybean 3-octenol could repel larval feeding and decrease their palmitic acid content. In conclusion, *C. bilineata tsingtauica* larvae are more adapted to R1 than to the other two soybeans. This study provides a theoretical basis for the production of higher-protein *C. bilineata tsingtauica* larvae.

## Figures and Tables

**Table 1:** The content (g/100g FW) of amino acids in different soybean cultivars.

Amino Acids	Soybean Varieties		
	Guandou-3 (G3)	Ruidou-1 (R1)	September Cold (SC)
Aspartic acid (Asp)	0.607 ± 0.007	0.727 ± 0.003	0.617 ± 0.003
Threonine (Thr)	0.247 ± 0.007	0.287 ± 0.003	0.253 ± 0.007
Serine (Ser)	0.243 ± 0.009	0.300 ± 0.000	0.243 ± 0.0120
Glutamic acid (Glu)	0.747 ± 0.023 a	0.655 ± 0.040 ab	0.580 ± 0.010 b
Glycine (Gly)	0.153 ± 0.003	0.187 ± 0.000	0.163 ± 0.003
Alanine (Ala)	0.193 ± 0.003	0.233 ± 0.008	0.205 ± 0.015
Cystine (Cys)	0.054 ± 0.002 ab	0.055 ± 0.002 a	0.045 ± 0.003 b
Valine (Val)	0.200 ± 0.010	0.223 ± 0.003	0.193 ± 0.007
Methionine (Met)	ND	ND	ND
Isoleucine (IIe)	0.103 ± 0.004 b	0.118 ± 0.003 a	0.110 ± 0.000 ab
Leucine (Leu)	0.340 ± 0.015	0.400 ± 0.011	0.365 ± 0.005
Tyrosine (Tyr)	0.263 ± 0.009	0.320 ± 0.006	0.263 ± 0.007
Phenylalanine (Phe)	0.127 ± 0.003 b	0.168 ± 0.009 a	0.135 ± 0.005 ab
Lysine (Lys)	0.397 ± 0.007	0.467 ± 0.003	0.400 ± 0.015
Histidine (His)	1.050 ± 0.006	1.023 ± 0.093	1.077 ± 0.044
Arginine (Arg)	0.193 ± 0.012	0.260 ± 0.006	0.200 ± 0.017
Proline (Pro)	0.127 ± 0.009	0.123 ± 0.012	0.120 ± 0.005

Note: Data are means ± SEM. ND indicates that the plant nutrient was not detected in soybeans. The lowercase letters in the figure indicate significant difference among different cultivars (*p* < 0.05, Tukey test).

**Table 2:** The content (g/100 g FW) of amino acids in *C. bilineata tsingtauica* larvae fed on different soybean cultivars.

Amino Acids	Larvae Treated with Three Soybean Varieties		
	Guandou-3 (G3)	Ruidou-1 (R1)	September Cold (SC)
Aspartic acid (Asp)	0.957 ± 0.032	0.950 ± 0.012	0.900 ± 0.070
Threonine (Thr)	0.457 ± 0.012 a	0.435 ± 0.016 ab	0.380 ± 0.000 b
Serine (Ser)	0.533 ± 0.050	0.577 ± 0.050	0.493 ± 0.028
Glutamic acid (Glu)	1.450 ± 0.015 a	1.328 ± 0.057 ab	1.190 ± 0.040 b
Glycine (Gly)	0.540 ± 0.038	0.560 ± 0.047	0.553 ± 0.029
Alanine (Ala)	0.630 ± 0.049	0.703 ± 0.042	0.647 ± 0.053
Cystine (Cys)	0.042 ± 0.0028	0.038 ± 0.002	0.031 ± 0.009
Valine (Val)	0.563 ± 0.037	0.587 ± 0.024	0.557 ± 0.043
Methionine (Met)	0.042 ± 0.004	0.043 ± 0.005	0.033 ± 0.004
Isoleucine (IIe)	0.367 ± 0.015	0.377 ± 0.003	0.367 ± 0.021
Leucine (Leu)	0.643 ± 0.024	0.673 ± 0.012	0.627 ± 0.040
Tyrosine (Tyr)	0.513 ± 0.006	0.543 ± 0.049	0.507 ± 0.064
Phenylalanine (Phe)	0.447 ± 0.018 a	0.380 ± 0.017 ab	0.345 ± 0.015 b
Lysine (Lys)	0.405 ± 0.171	0.543 ± 0.049	0.507 ± 0.062
Histidine (His)	0.560 ± 0.031	0.580 ± 0.015	0.520 ± 0.018
Arginine (Arg)	0.607 ± 0.033	0.620 ± 0.011	0.617 ± 0.034
Proline (Pro)	0.493 ± 0.049	0.497 ± 0.012	0.440 ± 0.031
Tryptophan (Trp)	0.098 ± 0.005 a	0.101 ± 0.003 a	0.078 ± 0.001 b

Note: Data are mean ± SEM. The lowercase letters in the figure indicate significant difference among different cultivars (*p* < 0.05, Tukey test).

**Table 3:** The content (g/100 g FW) of fatty acids in *C. bilineata tsingtauica* larvae fed on different soybean cultivars.

Fatty Acids	Larvae Treated with Three Soybean Varieties		
	Guandou-3 (G3)	Ruidou-1 (R1)	September Cold (SC)
Caprylic acid (C8:0)	ND	ND	ND
Decylic acid (C10:0)	ND	ND	ND
Undecanoic acid (C11:0)	ND	ND	ND
Lauric acid (C12:0)	ND	ND	ND
Tridecylic acid (C13:0)	ND	ND	ND
Myristic acid (C14:0)	ND	ND	ND
Myristoleic acid (C14:1)	ND	ND	ND
Pentadecanoic acid (C15:0)	ND	ND	ND
Pentadecenoic acid (C15:1)	ND	ND	ND
Palmitic acid (C16:0)	0.037 ± 0.004 b	0.072 ± 0.011 a	0.074 ± 0.007 a
Palmitoleic acid (C16:1)	ND	ND	ND
Margaric acid (C17:0)	ND	ND	ND
Margaroleic acid (C17:1)	ND	ND	ND
Stearic acid (C18:0)	0.034 ± 0.008	0.047 ± 0.004	0.047 ± 0.008
Elaidic acid (C18:1n9t)	ND	ND	ND
Oleinic Acid (C18:1n9c)	0.008 ± 0.001 b	0.008 ± 0.002 b	0.018 ± 0.003 a
Linolelaidic acid (C18:2n6t)	ND	ND	ND
Linoleic acid (C18:2n6c)	ND	ND	ND
Arachidic acid (C20:0)	0.007 ± 0.003	0.005 ± 0.002	0.005 ± 0.002
γ-Linolenic acid (C18:3n6)	ND	ND	ND
α-Linolenic acid (C18:3n3)	ND	ND	ND
cis-11-Eicosenoic acid (C20:1)	ND	ND	ND
Heneicosanoic acid (C21:0)	ND	ND	ND
cis-11,14-Eicosatrienoic acid (C20:2)	ND	ND	ND
Behenic acid (C22:0)	0.012 ± 0.003	0.010 ± 0.000	0.009 ± 0.000
cis-8,11,14-Eicosatrienoic acid (C20:3n6)	ND	ND	ND
cis-11,14,17-Eicosatrienoic acid (C20:3n3)	ND	ND	ND
Erucic acid (C22:1n9)	ND	ND	ND
Arachidonic acid (C20:4n6)	ND	ND	ND
Tricosanoic acid (C23:0)	ND	ND	ND
cis-13,16-Docosadienoic acid (C22:2)	ND	ND	ND
Eicosapentaenoic acid (C20:5n3)	ND	ND	ND
Lignoceric acid (C24:0)	ND	ND	ND
Nervonic acid (C24:1)	ND	ND	ND
cis-4,7,10,13,16,19-Docosahexaenoic Acid (C22:6n3)	ND	ND	ND

Note: Data are means ± SEM. ND indicates that the plant VOCs were not detected in soybeans. The lowercase letters in the figure indicate significant differences among different cultivars (*p* < 0.05, Tukey test).

**Table 4:** The relative content of plant volatile organic compounds (VOCs) in the three soybean varieties.

Classification	Name of Volatiles	CAS Number	Guandou-3 (G3)	Ruidou-1 (R1)	September Cold (SC)
Aldehydes	Benzaldehyde	000100-52-7	0.0040 ± 0.0002	0.0035 ± 0.0003	0.0042 ± 0.0004
	Nonanal	000124-19-6	0.0094 ± 0.0003	0.0087 ± 0.0009	0.0144 ± 0.0008
	Heptadienal	004313-03-5	0.0112 ± 0.0017	ND	0.0151 ± 0.0018
	Salicylaldehyde	000119-36-8	ND	0.0207 ± 0.0009	0.0297 ± 0.0056
	2,4-octadienal, (E,E)-	030361-28-5	ND	0.0027 ± 0.0002	ND
	Heptanal	000111-71-7	ND	ND	0.0028 ± 0.0003
Esters	Methyl salicylate	000119-36-8	0.0165 ± 0.0021	0.0625 ± 0.0041	0.0213 ± 0.0016
	Dihydroactinidiolide	015356-74-8	ND	0.0065 ± 0.0003	ND
Alcohols	3-octenol	003391-86-4	0.5364 ± 0.0081	0.4902 ± 0.0044	0.5109 ± 0.0147
	1-hexanol	000111-27-3	0.0130 ± 0.0011	ND	ND
	1-penten-3-ol	000616-25-1	ND	0.0104 ± 0.0008	0.0087 ± 0.0009
	trans-2-pentene	001576-87-0	ND	0.0048 ± 0.0011	0.0036 ± 0.0007
	2-penten-1-ol, (Z)-	001576-95-0	ND	ND	0.0080 ± 0.0009
Ketones	3,5-octadien-2-one	038284-27-4	ND	ND	0.0077 ± 0.0020
	5, 6-epoxy-β-ionone	023267-57-4	0.0133 ± 0.0015	0.0176 ± 0.0005	0.0182 ± 0.0025
Heterocyclic compound	Furan, 2-ethyl-	003208-16-0	0.0211 ± 0.0016	0.0166 ± 0.0004	0.0230 ± 0.0017
	Furan, 2-pentyl-	003777-69-3	0.0063 ± 0.0009	0.0085 ± 0.0015	0.0090 ± 0.0013

Note: Data are means ± SEM. ND indicate that the plant VOCs were not detected in soybeans.

## Data Availability

The data presented in this study are available on request from the corresponding author.

## Supplementary Materials

The following supporting information can be downloaded at: https://www.mdpi.com/article/10.3390/foods12081721/s1, Figure S1: The standard curve for tryptophan (Trp) measurements.
