# Supplementary figures and images for: Nutritional and Feeding Adaptability of Clanis bilineata tsingtauica Larvae to Different Cultivars of Soybean, (Glycine max)
Source: Foods. 2023 Apr 20;12(8):1721. doi: 10.3390/foods12081721 (PMC10137361; doi:10.3390/foods12081721)

## Supplementary Materials

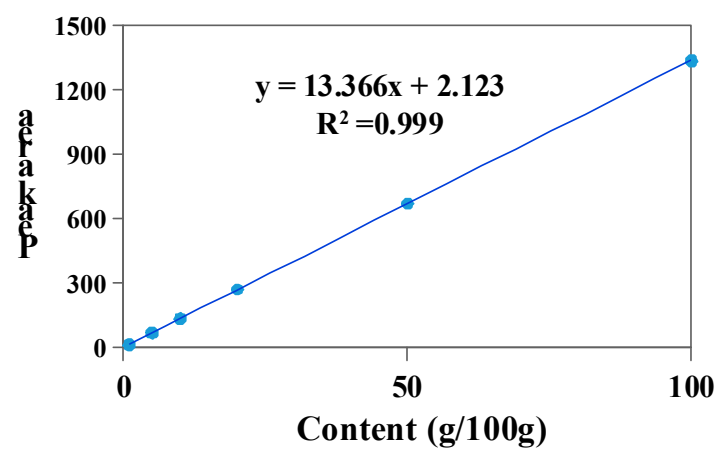

**Figure S1** The standard curve for tryptophan (Trp) measurements

Supplement: Supplementary file 1 [file foods-12-01721-s001.zip › foods-2253484-supplementary.pdf]
